# Elevated Concentrations of Metal(loids) in Seaweed and the Concomitant Exposure to Humans

**DOI:** 10.3390/foods10020381

**Published:** 2021-02-10

**Authors:** Mayeen Uddin Khandaker, Nwokoma Oliver Chijioke, Nurul’ Adillah Binti Heffny, David A. Bradley, Abdullah Alsubaie, Abdelmoneim Sulieman, Mohammad Rashed I. Faruque, M. I. Sayyed, K. S. Al-mugren

**Affiliations:** 1 Centre for Applied Physics and Radiation Technologies, School of Engineering and Technology, Sunway University, Bandar Sunway 47500, Malaysia; oliveryoung84@gmail.com (N.O.C.); d.a.bradley@surrey.ac.uk (D.A.B.); 2 Department of Physics, University of Malaya, Kuala Lumpur 50603, Malaysia; dillaheffny@siswa.um.edu.my; 3 Department of Physics, University of Surrey, Guildford GU2 7XH, UK; 4 Department of Physics, College of Khurma, Taif University, P.O. Box 11099, Taif 21944, Saudi Arabia; a.alsubaie@tu.edu.sa; 5 Department of Radiology and Medical Imaging, College of Applied Medical Sciences, Prince Sattam Bin Abdulaziz University, P.O. Box 422, Alkharj 11942, Saudi Arabia; a.sulieman@psau.edu.sa; 6 Space Science Centre (ANGKASA), Universiti Kebangsaan Malaysia, Bangi 43600, Malaysia; rashed@ukm.edu.my; 7 Department of Physics, Faculty of Science, Isra University, Amman 11622, Jordan; mabualssayed@ut.edu.sa; 8 Department of Nuclear Medicine Research, Institute for Research and Medical Consultations, Imam Abdulrahman bin Faisal University, Dammam 31441, Saudi Arabia; 9 Department of Physics, Princess Nourah Bint Abdulrahman University, Riyadh 11144, Saudi Arabia

**Keywords:** seaweeds, ICP-OES, metal(loids), marine pollution, non-carcinogenic & carcinogenic risks

## Abstract

While the consumption of seaweed and seaweed-based products is very common amongst East Asian nations, forming a notable component of the daily diet, relatively very few studies have concerned the concentrations of heavy metals in these together with potential effects on human health. The present study analyses the concentrations of 17 elements in locally resourced seaweed, also assessing potential noncarcinogenic and carcinogenic risks. The samples were ground, homogenized, and quantified using the ICP-OES technique. It has been found that the essential elements K, Ca, Mg, Zn, and Na typically show concentrations somewhat greater than a number of potentially toxic metals, in particular, Cd, Pb, Ag, and As, with exceptions being Ni, Cr-VI, and Si. Statistical analysis indicates all of the latter to have similar origin, with increased concentration of these metals within the marine ecosystem. While the daily estimated intake of most metals is seen to be within the daily dietary allowance level recommended by various international organizations, the noncarcinogenic risk shows a value greater than unity, estimated via the hazard quotient. This indicates a potential for adverse effects to health arising from consumption of the sampled seaweed. The carcinogenic risk resulting from nonessential elements shows values greater than the United States Environmental Protection Agency (US-EPA) reference limit of 10^−4^. Considering the nonbiodegradability of heavy metals and metalloids and their potential accumulation in seaweed, there is need for critical examination of metal levels in the seaweeds obtained from the present study locations, together with the introduction of practices of removal of heavy metals via bio-adsorbent techniques.

## 1. Introduction

Seaweeds or macroalgae, plant-like organisms, usually refer to the several species of multicellular marine algae. These are classified into three categories: red algae (Rhodophyta), brown algae (Phaeophyta), and green algae (Chlorophyta). While the red algae are multicellular, only a large part of brown algae is multicellular, with both being principally of marine origin. Green algae are largely unicellular and nonmarine [1]. Red, brown, and green seaweeds are generally distributed in the subtidal, tidal, and intertidal regions, respectively. From the earliest times of human habitation, seaweed has formed a part of the human dietary chain, appearing both directly within food and also as a result of indirect utilization as feed, fodder, and manure. Some species of edible seaweed contain significant amounts of minerals, vitamins, proteins, fibers, carbohydrates, macronutrients (N, P, K, Ca, Mg, and S), and micronutrients (Zn, Cu, and Mn), are low in fat and calorific value, and also possess important biological compounds that are known to help combat disease [2,3]. The red algae find considerable use in modern European and Asian cuisine, also being used to make other products such as agar, carrageenans, and food additives. Due to the multicellular characteristics of macroalgae, the cell wall comprises a variety of polysaccharides and proteins, some containing sulfate, anionic carboxyl, and phosphate groups that have a very high capacity for metals retention [4]. Indeed, seaweeds are recognized to retain metals, many times more concentrated than those prevailing in the surrounding waters [5,6]. Accordingly, the implication is that several organisms in the aquatic system can bio-accumulate the various groups of metals in a different way, eventually appearing within the human gut microbiome via the consumption of seafood [7]. Clearly, the concentration of metals in seaweed is modified by the concentrations in seawater, biota, and sediments. 

In recent times, rapid growth in urbanization and large-scale industrial activities throughout the world have contributed immensely to increasing metals contamination in the aquatic system [8]. Of particular concern are the heavy metals and metalloids from anthropogenic activities, including mining, milling, petrochemicals processing, printing, the electronics industry, and municipal waste, be it directly discharged into the marine environment or transported into the greater aquatic system via estuaries [9]. Accordingly, once toxic metals are introduced into the aquatic systems within which seaweeds are cultivated or grow naturally, they are easily accumulated in the multicellular marine macroalgae. In this, it is also to be appreciated that accumulation of metals in seaweed results not from anthropogenic sources alone but is also contributed to by the various natural factors that impact upon the marine ecosystem (volcanic activities and tsunami occurrences included) [5,10,11]. Metals are accordingly accumulated in the food chain, beginning with uptake at the primary producer level, subsequently at the consumer level, finding their way into the body. Despite the significant nutritional value of seaweed, considerable concern surrounds the potential adverse health effects from seaweed consumption, specifically in regard to exposure to heavy metals and other chemical toxicants. Instances of toxicity arise even at trace levels of 0.05 to 1 mg kg^−1^, including from heavy metals such as mercury (Hg), arsenic (As), cadmium (Cd), and lead (Pb), with their presence in the body posing potential carcinogenic effects [6,12]. The toxicity of metals can be further linked with interference to the normal function of the enzymes, and with deleterious effects to the haemopoietic, nervous, urinary, gastric, and genital systems [13]. 

In regard to the present study, it is to be anticipated that the aquatic environment of Malaysia receives nonnegligible amounts of metal and metalloids loadings, deriving from multimodal anthropogenic activities, including sea transportation, not least tanker traffic, oil-and-gas exploration, power plant and other coastal industrial operations, agricultural undertakings, and the associated waste streams of urbanization [14]. While there is a compelling need to assess the environmental impact, it is apparent that only a few studies have pointed to the heavy metal pollution incurred by the terrestrial [14,15,16] and marine environments in Malaysia [8,17]. As an instance, Khadijeh et al. [8] detected a considerable level of As, Cd, Cr, Cu, Hg, Ni, Pb, and Zn in surface sediments of the coastal areas of the South China Sea. In performing a study of the Malaysian aquatic environment, Noor et al. [17] reported that the Langat and Juru Rivers were contaminated by Pb and Zn, while the Langat River also showed elevated concentrations of Cd. The concentrations of Zn and Pb in coastal sediments of the Juru River were reported to be 2–3 times greater than global shale values. Noor et al. [18] studied the metal levels in sediment and seawater from coastal area of the Strait of Malacca, reporting a higher concentration of metals in bottom seawater samples compared with surface seawater, and a significant enrichment of Cd, Cu, Zn, and Pb in the sediment samples. Note that although, in general, no information has been given concerning the depth of seawater sampling points, a maximum depth of 13 cm was reported for the sediment samples. While no earlier studies on metal levels in seaweeds are available in Malaysia, it is further apparent that relatively few studies are to be found from other parts of the world. In this regard, Pan et al. [19] observed the high bioaccumulation capability of specific heavy metals (Cu, Cr, Ni, Zn, Pb, Cd, and As) by seaweed collected from the Dongtou Islands of the East China Sea. Karthick et al. [20] reported typical concentrations of Mn, Pb, Zn, Cd, Cu, and Cr in six types of South Andaman Island seaweed, an island situated in the Bay of Bengal. Dadolahi-Sohrab et al. [21] reported the presence of the metals Pb, Cd, Cu, Ni, Zn, and Fe in 11 dominant seaweed species from the Strait of Hormuz, with concentrations greater than that from other different regions of the world. Malea et al. [22] reported relatively high concentrations of Fe and Pb in green and brown seaweeds from the Antikyra Gulf (Viotia, Greece), while Bryan and Hummerstone [23] measured the concentrations of Zn, Mn, Fe, Pb, and Cu in macroalgae collected within the region of estuaries in the south-west of England. Mutia and Mtolera [24] reported greater concentrations of As, Cd, and Hg in seaweed from Kenya than in plants in the terrestrial system, due to the redistribution of terrestrial materials within the ecosystem. They also reported very high concentrations of total as (103–147 mg kg^−1^ dry weight, dw) and inorganic as (32–70 mg kg^−1^ dw).

Given that among the marine organisms accumulating both essential and nonessential elements, there are those that absorb in proportion with their environmental concentrations, contamination of coastal environment by anthropogenic activities can be monitored with the help of such indicator organisms. Among the various organisms, seaweeds offer an important function in monitoring of the nutrient dynamics of coastal biosystems, efficiently reflecting changes in water quality [25]. Thus said, while seaweeds have been widely used to assess the health of coastal environments, characterizing, and monitoring the status of environmental pollution, the determination of metal/metalloids in seaweeds has received little attention. To the best of our knowledge, within the coastal region of Malaysia, there is an absence of data on heavy metals in seaweed. While the largest production percentages of seaweeds are from other East Asian countries, from the 1970s onwards Malaysia has also made considerable efforts in cultivating seaweed, recognizing the potential for high economic returns. In this regard, in order to protect the consumer, regular surveys of toxic metals in seaweeds and estimation of health risk need to be considered. Considering that consumption of food is the major source of heavy metals exposure/bio-accumulation in the body, this study has sought to assess the levels in seaweed of the metals ^19^K, ^20^Ca, ^12^Mg, ^82^Pb, ^48^Cd, ^34^Se, ^13^Al, ^25^Mn, ^29^Cu, ^30^Zn, ^26^Fe, ^33^As, ^11^Na, ^28^Ni, ^24^Cr, ^47^Ag, and ^14^Si, with use being made of inductively coupled plasma optical emission spectrometry (ICP-OES). An additional aim was to estimate the carcinogenic and noncarcinogenic risks of metals exposures to the Malaysian population/consumers.

## 2. Cultivation Practices of Seaweed Plants in Malaysia

Malaysia provides a favorable habitat for the growth of marine macroalgae, noting the extensive coastline, moderate temperature (20–30 °C), abundant elevated levels of solar radiation, and equitable seawater salinity (30–35 ppm) and pH (see below), with numerous islands having sandy sea-beds with corals. By virtue of its location in the equatorial zone, throughout the year Malaysia receives rather elevated levels of solar radiation, averaging out at between 4000 and 5000 Wh/m^2^ day^−1^, with abundant sunshine for some 12 h day^−1^ [26]. In regard to salinity, this represents the total amount of dissolved salts per kg of seawater, and is expressed in terms of parts per million (ppm) or alternatively as microgram (μg) of dissolved salt per gram of seawater. In general, the weight ratio of the different types of dissolved salts in seawater is similar across the waters of the world’s oceans. At present, salinity is typically assessed by measuring the electrical conductivity, the salinity determined via this approach being a dimensionless quantity, with practical salinity in the scalar range 0.5 to >65. Based on such a scale, typical seawater has a salinity of 35 ppm (http://www.coastalwiki.org/wiki/Salinity, accessed on 24 February 2021). Noting the growing demand for seaweed-based commodities among East Asian and European countries, seaweed cultivation first took place in Malaysia in 1978, in Sabah. Medium-to-large-scale seaweed farms have since been developed in coastal areas of the South China Sea (Semporna, in particular Kota Belud) and the Straits of Malacca (in Langkawi). Principally, there are two species of seaweeds that are commonly found in these locations: *Eucheuma denticulatum* (red algae) and *Eucheuma cottoni* (various colors algae). These and other species are also to be found in various other locations around Malaysia. 

Within the Pulau Dangli area of Langkawi (a Straits of Malacca island with extensive coastline), seaweed farming is carried out on a somewhat smaller scale than that in east coast Semporna, Sabah; while several species of seaweed have been planted only one species (*Eucheuma cottoni*) has adapted strongly to the aquatic environmental conditions. Seaweed farming has also developed, albeit at a slower pace, in a number of other Sabah regions, such as Lahad Datu and Kunak on the east coast, and Kudat and Kota Belud on the west coast, all parts of the coastline of the South China Sea. The seaweed species *Eucheuma denticulatum*, cultivated within the west coast of Sabah, finds principal use for medicinal purposes, relating specifically to disease resistance. The cultivation methods vary, with both onshore and offshore activities. Prior to the period of cultivation, young and healthy seedlings (200 to 250 g) are harnessed with plastic ropes and then hung from a principal set of ropes, subsequently being planted in isolated marine waters at a tidal height of 40–70 cm on rocky and sandy sea-beds [1]. In accord with the sea depth, suitable methods of cultivation are chosen, including the stake, long-line, and raft system (each using the basket method). Recently, the farmers of Semporna have started to make use of shortened farming lines, offering less likelihood of the waves breaking-up the seaweeds, also facilitating ease and frequent management, pointing to increased farm productivity. Within a period of one to two months from planting, the mature seaweed can typically be harvested, conducted either by untying lines or by cutting the raffia ties from longlines. These are then carried to a main drying platform (concrete slabs or wooden platforms) and hung-out using elevated drying racks. At the platform, the culled seaweeds are graded/identified by the farmers, either to be dried or to be used as seedling material for the next production cycle. The drying process typically takes 3–5 days to ensure a moisture-free condition. The dried samples are then ready for further processing.

## 3. Materials and Methods

### 3.1. Sample Collection and Preparation

**Figure 1:**
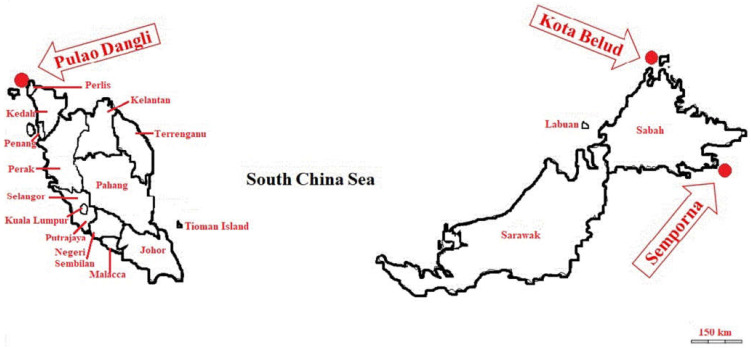
The locations of seaweed farming and sampling under this study.

Fresh seaweed samples (*Eucheuma cottoni*) were collected from the major seaweed farming locations in east and west Malaysia. A sampling campaign was carried out in March 2018, with samples collected from Kota Belud (6.3500° N, 116.4333° E) and Semporna (4°28′59.99″ N, 118°36′59.99″ E); in the same year, in September, further sampling took place in Pulau Dangli (6.44972° N, 99.77694° E). An amount of 4 kg of fresh seaweed (whole) was collected from each of the locations as show in Figure 1. The samples were obtained from at least four sublocations at each farming site in order to avoid any bias and also to ensure representative sampling of seaweeds within a particular farm. The collected samples were then transferred to separate plastic bags and brought to the laboratory. The collected seaweeds were washed to ensure the removal of all foreign media, including sediment, detritus, mud, unwanted weeds, and grazers. Using a clean stainless-steel knife, the samples were then cut into smaller pieces, ensuring sample surfaces were well exposed to air to facilitate effective drying and subsequent grinding. To remove remnant moisture, the samples were dried over the period of a day using a programmable closed system microwave oven maintained at 70 °C, the material being brought to a constant dry weight. A heavy-duty blender was then used to blend the samples, homogeneity subsequently been ensured by sieving using a 25 μm mesh sieve. The samples were then transferred into individually marked beakers/containers and retained at room temperature (26 °C) prior to chemical analysis.

### 3.2. Chemical Analyses by ICP-OES

In this study, all acids and chemicals were of analytical grade. Moreover, all equipment and glassware used for the analyses were carefully washed using 10% HNO_3_ solution, finally being doused in deionized water and then air-dried prior to use. An exact amount of 0.5 g dw from each sample was placed into a Teflon vessel. The samples in the vessel were digested via addition of 10 mL of HNO_3_ and 3 mL of HCl, then heated by placing onto a hot plate at 100 °C for 2 h (until only a small amount of liquid remained at the bottom of the beaker). The solution was then allowed to cool to room temperature, subsequently being filtered via use of a glass funnel containing Whatman No. 41 filter paper (0.8 μm pore size). In a volumetric flask, the filtered solution was further diluted by adding 10 mL of deionized water and stored at 4 °C, ready for analysis through use of inductively coupled plasma optical emission spectroscopy (ICP-OES, Model Optima 5300DV, PerkinElmer, Inc. Waltham, MA, USA). Blanks and standards were also prepared using the same volume and acid combinations, also adopting the same procedure as that used in preparation of the seaweed samples [1]. Dry weight concentrations of the various elements of interest were determined from calibration curves for the standards, with additional use of the following Equation (1):

(1) $$Metal\;concentration\;\left(\frac{\mathrm{mg}}{\mathrm{kg}}\right)=\frac{R_{ICP\;}\left(\mathrm{ppm}\right)\times \;V_{final\;}\left(\mathrm{mL}\right)\times \;D_{factor}}{W_{initial}\;\left(g\right)}$$

*R_ICP_* being the ICP-OES reading in ppm (=mg/L), *V_final_* the final volume of the sample after digestion (mL), *D_factor_* the dilution factor based on the ratio *V_final_* to *V_aliquot_* (from the aliquot amount of sample taken for ICP-OES analysis), and *W_initial_* the initial weight of the sample (grams). Considering instrumental accuracy, while use has been made of the procedure described in our previous study [16], some salient features from the present study need explanation. In particular, instrument calibration made use of a multi-element calibration standard solution 2A (for each element, starting with 10 mg/L stock solution; Agilent Technologies, Santa Clara, CA, USA, part no. 8500-6940) in 5% pure HNO_3_, diluted to within the range 10 mg/L to 100 mg/L. In obtaining an analysis of each sample, use was made of one blank solution and five standards, the same reagents being used under the same conditions as those used for the control, seeking to avoid contamination from the digestion procedures. In validating the ICP-OES results, for each ICP-OES run, use was made of National Institute of Standards and Technology (NIST) standard reference material SRM 1400 and National Metrology Institute of Japan (NMIJ) CRM 7405-a. Results showed greater than 96% recovery values for all of the analyzed metals, comparison being made against certified values (See in Table 1). Determination of the limit of detection (LoD) has exploited both the measured limit of the blank (LoB) as well as test replicates of a sample known to contain a low concentration of the analyte [27]. The LoD was defined using the expression 3 × SD_blank_/M, where SD_blank_ is the standard deviation of at least 3 replicate measurements of the blank, and M represents the slope of the calibration curve. The calculated LoD values are presented in Table 2. For concentrations of the metals of interest, each sample was analyzed in duplicate. 

### 3.3. Assessment of Health Risk

In consumption of contaminated foodstuffs, the United States Environmental Protection Agency (US-EPA) has developed models to calculate the detrimental impacts of heavy metals on human health. Accordingly, the present study has evaluated the health risks associated with the consumption of metals-contaminated seaweed collected from the defined areas. Assessment of the associated health risks were based on the daily intake (DI) of each metal and other pertinent parameters (for instance, body weight). 

### 3.4. Daily Intake (DI) of Heavy Metals

For a given population, the estimation of daily intake of analyzed metals (in microgram per kilogram per day (μg/kg/day) depends strongly on the concentration of metals in the sample and consumption details for the seaweed. The DI of metals for adults was calculated using the following Equation (2):

(2) $$\mathrm{DI}=\frac{C_{\mathrm{metal}}{\;\times \;C}_{R}\;}{\mathrm{BW}}$$

with C_metal_ the mean metals concentration (mg/kg dw) in each sample, C_R_ the consumption rate, and BW the average body weight for an adult population, considered to be 70 kg [14]. Year 2018 Malaysian fisheries sector data for capture fisheries and aquaculture show respective production of 1.48 million ton and 397,000 metric ton, macroalgae (seaweed) being the major aquaculture contributor (31% by weight) [28]. The same study reported the export of the majority of seaweed produced in Malaysia to East Asian countries, including China and the Philippines. Over the last 40 years, daily seaweed consumption per person in Japan has grown by a relatively small degree, from 4.3 g/day in 1955 to 5.3 g/day in 1995 [29], compared to a far greater increase in seaweed consumption by adults from South Korea, from 8.5 g/adult/day in 2010 [30] to 25.3 g/adult/day in 2018 (https://www.statista.com/statistics/1047839/south-korea-daily-seaweed-consumption-per-capita/, accessed on 10 December 2020). For a Chinese population, Chen et al. [31] reported a seaweed consumption rate of 5.2 g/capita/day, similar to that for Japan. Noting also that the use of seaweed ranges from inclusion in cosmetics to that in foodstuffs, a daily intake of 10 g/adult/day has been adopted for an average adult East Asian, used herein in estimating metals loading and health risk. One further factor to be considered for estimating the daily intake of metals is the use of 65 kg as the nominal body weight for East Asian nations.

### 3.5. Estimation of Mean Daily Dose (MDD)

The mean daily dose (MDD) (mg kg^−1^ day^−1^) provides an appraisal of the level of metal exposure to humans due to the consumption of seafood/seaweeds, and determined by the Equation (3):

(3) $$\mathrm{MDD}=\frac{{\mathrm{DI}\;\times \;E}_{f}{\;\times \;D}_{e}}{T_{p}}$$

with E_f_ the exposure frequency (250 days/year), based on local knowledge of seaweed consumption, assumed at 5 days per week, and D_e_ being the lifetime period of exposure to the ingested metals. Considering the average lifespan of Japanese, Korean, and Chinese, of 84, 82, and 76 years, respectively, a nominal value of 75 years has been used for the duration of ingested metals exposure (neglecting several years at the beginning and end of life). T_P_ is the time period over which the dose has been averaged (in days), generally considered equal to D_e_ for noncarcinogenic effects (=365 days × D_e_). Considering the inherent toxicity of heavy metals, the MDD provides a basis in estimating noncarcinogenic and carcinogenic risk effects to human health from such exposures.

### 3.6. Noncarcinogenic Risk

The hazard quotient (HQ), characterizing the noncarcinogenic risk to an exposed individual due to a single metal, is defined as the ratio of the mean daily dose of a particular metal to the reference dose (R_f_D), with the latter a noncarcinogenic threshold for that metal toxicant (US-EPA, 1993). Non-carcinogenic health risk arising from metal exposure (via the oral pathway) can be estimated by using Equation (4) as follows:

(4) $$\mathrm{HQ}=\;\frac{\mathrm{MDD}}{R_{f}D}$$

The R_f_D values for most of the toxicants (typically quoted in mg/kg/day) have been set out by a number of international bodies, including the WHO, Office of Environmental Health Hazard Assessment (OEHHA), and US-EPA [32,33,34,35,36], as well as in [37]. The sum of HQs for all major metals provides the estimated potential for adverse effects from exposures to multiple metals, assuming each to have similar working mechanisms and linear effect upon a given target organ [38,39]. Generally, it is considered that an exposed population is at minimal risk when the hazard index is (HI = Σ HQs) < 1 [38,39,40].

### 3.7. Carcinogenic Risk

In regard to carcinogenic risk, this can be characterized by a linear association between the intake dose of carcinogenic metals and concomitant effects [41]. To a population exposed to potential carcinogens within consumed seaweed, the lifetime cancer risk (LTCR) has been estimated in accord with the following Equation (5):

LTCR = MDD × CSF × LT
(5)

where the carcinogen potency factor, CSF is the cancer slope factor, representing the upper-bound estimate of the slope of the dose–response curve in the low-dose region [34,42]. The LT is the average lifetime of the respective population consuming the seaweeds (considered herein to be 75 years). The life time cancer risk (LTCR) was determined for several of the metals (Cd, As, Cr, Ni, and Pb), oral exposure being considered to bear carcinogenic risks. The cumulative cancer risk arising from exposure to metal carcinogens in consumed foodstuffs is assumed to be a linear sum of each of the individual metal risks [40,43,44], and can be obtained by Equation (6):

(6) $$\sum_{i=0}^{n}=\mathrm{LTCR}1\;+\;\mathrm{LTCR}2+\;\ldots \ldots \;+\;\mathrm{LTCR}n_{\;\;\;}$$

with *n* = 1, 2 …… *n* representing the individual metal contaminants/carcinogens in foodstuffs. Equation (6) gives the totality of carcinogenic risk of all the heavy metals present in the seaweed. While risk assessment models are invariably subject to uncertainties and the need for refinements as more data become available, they nevertheless offer a basis from which the relationship between human health and metal toxicity can be estimated, both carcinogenic and noncarcinogenic, with effects quantified through the exposure framework [45,46].

## 4. Results and Discussion

### 4.1. Concentrations of Metals in SEAWEEDS

Table 3 shows the measured concentrations of metals in the studied seaweed samples collected from three major seaweed farms in Malaysia. The data are expressed as mean ± relative standard deviation (RSD), use being made of the Microsoft Excel software. As expected, concentrations of essential mineral elements show relatively greater values than the potentially toxic metals. Cu is one of the essential elements showing a low concentration, appearing at a level similar to that of Ag, Cd, and As. While in the sampled seaweeds the essentiality of Cu is only provided for at lower levels, it is nevertheless considered to be above that at which low-Cu effects in the body might occur. Conversely, the highest value amongst all of the metals analyzed in this study is that for the electrolytes, K, followed by Na, Ca, and Mg. Table 3 also shows a general comparison of the currently measured data with available literature values from other locations around the world. Measured data for all of these essential mineral elements, such as Zn, Mg, Fe, Mn, Ca, and Na, are typically in line with data of the available literature (see in Table 3). The presence of considerable amounts of these metals indicates that seaweed forms a good source of nutrients for human health. On the other hand, although the measured data of potassium are in line with those reported by Smith et al. [3] and Ruperez [47], they are much greater in value than the typical 380 ppm (i.e., µg/g) in seawater (https://web.stanford.edu/group/Urchin/mineral.html, accessed on 4 December 2020). Nevertheless, potassium is excreted through the urine, also being under strict homeostatic control in the healthy individual. The concentrations of some nonessential metals, Ag, Cd, and As, are some several orders of magnitude lower than that of the other metals studied herein (see Table 3). The observed variation in metal concentrations in samples collected from different locations is reflective of the various anthropogenic factors at work, including that of the farming environment, sometimes with profligate heavy metals/elements introduced into the aquatic ecosystem. A brief discussion on the potential toxic elements is presented here.

Chromium (Cr) can exist in several oxidation states from 0 to 6+. Due to its lower solubility, the trivalent Cr (III) in plants is found to be much lower than the hexavalent chromium (VI). The toxicity of chromium to the human body is mainly attributable to the absorption of Cr (VI) within the lung and gastrointestinal tract [54], and even to a certain extent by intact skin [55]. Again, high rates of Cr (VI) can cause several bio-toxic effects, including to the hepatic, renal, and hematological systems [56,57]. Table 3 shows the highest mean concentration of Cr-VI, at 40.57 μg/g in Langkawi (LKW) samples, followed by 40.15 μg/g in Kota Belud (KBL) and 30.3 μg/g in Semporna (SPN). The present study shows consistency with the recent data reported by Chen et al. [31] but are considerably greater than the data reported by Lorenzo [48] and Smith et al. [3]. This may reflect environmental contamination arising from various anthropogenic activities. 

Pb toxicity affects the systems and organs of the human body [44,58]. Even low intake of Pb (3.0 μg/kg/day) can cause permanent damage, including reduced IQ, learning disabilities, and shortened attention span [54,59]. In our study, the KBL and SPN samples showed the greatest and least concentrations at 10.85 ± 1.16 μg/g and 3.75 ± 4.56 μg/g, respectively. Table 3 shows our data to be in agreement with the literature data reported by Lorenzo [48], Chen et al. [31], and Malea and Haritonidis, [51], but greater than the reported levels of Smith et al. [3], Akcali and Kucuksezgin [50], and Al-Masri et al. [52], and lower than that reported by Khaled et al. [49] and Schintu et al. [53]. There is an anticipation that the Malaysian marine environment, criss-crossed by major shipping lines, contains a nonnegligible amount of Pb, not least in the Kota Belud and Langkawi areas.

Cadmium (Cd) is known as a priority environmental pollutant and a nonessential element for human health [60]; high ingestion is associated with lung damage, renal damage, and skeletal changes [61,62]. Inconsiderable variation in Cd level is found among the present studied locations. Our measured data are also within the range of most of the literature data, albeit with Schintu et al. [53] reporting an elevated concentration of Cd in seaweed collected from south-western Sardinia, Italy. 

Naturally, arsenic (As) can exists in various chemical forms: inorganic arsenic species like As (III) and As (V), and organic arsenic species like arsenobetaine (methylarsonate, MMA) and arsenosugars (dimethylarsinate, DMA) [45]. The toxicity of inorganic arsenic As (III) and As (V) is about 100 times greater than that of monomethylarsonic acid (MMA) or dimethylarsinic acid (DMA). It is worth mentioning that these arsenic species can be separated by using anion-exchange HPLC conditions, and the ICP-OES measurements usually provide the elemental ^75^As rather than a particular species. Zhao et al. [45] studied the various species of arsenic in seaweed via HPLC and ICP-MS techniques, and finally reported only inorganic As(III) in their studied edible seaweed samples. Based on their study, the measured data in the current study are principally the inorganic As (III). It has been reported that even a low concentration of arsenic (0.5 μg/kg, dw) can cause carcinogenicity and teratogenicity [63]. Table 3 shows very high uncertainties in As levels in seaweed collected from SPN (1.6 ± 5.86 μg/g) and KBL (5 ± 7.75 μg/g), implying As to be more spread out in the relevant environment. The measured As values lie within the range of literature data of elsewhere. Comparing with the typical As concentration of 0.024 ppm in seawater, the present study indicates the appearance of As contamination in the studied locations.

It has been reported that excess intake of Se can lead to selenosis, symptoms including a garlic odour to the breath, gastrointestinal disorders, loss of hair, sloughing of nails, fatigue, irritability, and neurological damage [64,65]. Table 3 shows that LKW samples contain much lower levels of Se than those of SPN and KBL locations. A low level of Se (0.01–0.1 μg L^−1^) naturally exists in most freshwater and saltwater environments, but certain anthropogenic activities may increase Se loading into aquatic ecosystems [66]. This fact indicates that the coastal waters of South China Sea are receiving more Se from various anthropogenic activities than the Andaman sea. Overall, the measured data of Se show relatively higher levels than the literature values (Table 3). 

The metalloid silicon (Si) is largely used as an additive in the food and beverage industry [67]. However, the bioavailability of Si is occurred in the form of orthosilicic acid, whereas thin fibrous crystalline asbestos is a health hazard promoting asbestosis and significant impairment of lung function. The measured data of Si show a great variation among the studied locations. Silicon is a part of various minerals, from which it may be released during weathering processes. However, this study shows no comparison due to the lack of any literature data. There is no specified acceptable daily intake for this metalloid, however the Food and Drug Administration (FDA) of U.S [68] has set a maximum of 2% of SiO_2_ in a food’s total weight, while EU [69] sets a maximum level for silica of 1% by weight in dried powdered foodstuffs.

A high intake of Aluminum has been found to adversely affect the human reproductive and nervous systems, and shows a potential association with Alzheimer’s disease [70]. The mean level of Al in the samples from LKW are seem to be much above that of the other studied locations (Table 3). Nevertheless, present study values lie in the lower range of the reported data of Chen et al. [31], offering evidence in support of Malaysian seaweed posing no known Al toxicity.

Silver (Ag) presents no known physiological benefits when taken orally. The US-EPA identified this metal as a Group D carcinogen (i.e., not carcinogenic in humans) [71]. The notable toxic effects of Ag in humans come mostly from its salts, and include argyria, gastrointestinal irritation, and renal and pulmonary lesions [72,73]. Measured data for Ag show negligible variation among the studied locations. The present results show negligible contamination of seaweed by Ag in comparing with the literature data reported in ref [48] and also the intake limit of 0.005 mg/kg/day recommended by the US-EPA [71].

Table 4 presents projected metal daily intakes in microgram per gram/kg/day for a typical East Asian consumer arising from exclusive consumption of Malaysian seaweed within the diet, together with recommended total dietary intake levels. For the essential element K, the projected mean intake far exceeds the recommended level of 3510 μg/kg/day [74]. Other than this, the estimated daily intakes for all of the other studied metals are lower than the respective recommended levels for metal intake. Such intake levels would typically not represent human health concern, the recommended values being for total dietary intake while seaweed forms a minor contribution to the total dietary habit. Nevertheless, since in foodstuff the presence of toxic metals (Pb, Cd, Cr, and As) is generally considered undesirable, even at the projected levels, reduced/controlled consumption of seaweeds may well help to avoid risks to human health. 

### 4.2. Health Risk of Metal Exposures via the Consumption of Seaweeds

Since seaweeds have greater metals bioaccumulation capability than the majority of other aquatic plants/organisms, their consumption may create a degree of risk. Table 5 shows the estimated noncarcinogenic and carcinogenic risks due to ingested metal exposures arising from the consumption of Malaysian seaweeds. Since the seaweeds that have been investigated herein are mostly exported to East Asian countries, the concomitant risks are calculated using pertinent data for the populations of that region. The calculated hazard quotient (HQ) values for Cd, Cr-VI, Se, As, and Mg all indicate non-negligible chemical toxicity. The total hazard index (HI) represents noncarcinogenic (chemical) risk due to the exposure from multiple metals, being found to be 4.38. That it is substantially greater than 1 indicates it to be at the level of major concern. The metals As, Cd, Ni, Cr-VI, and Pb are all known to be potential carcinogens [82,83], the LTCR due to exposure to these from lifetime consumption of seaweeds for the East Asian population being summarized in Table 5. Here, it should be mentioned that the so-called heavy metal ions can result in carcinogenic outcomes, a result of their interactions with and the damage caused to DNA and nuclear proteins [84]. In 2017, the WHO urged Member States, “to implement comprehensive cancer prevention and control programs, including management of disease […] fostering the development of effective and affordable new cancer medicines” also, “to enhance the coordination of activities related to the assessments of hazards and risks and the communication of those assessments” [85]. Therefore, a more practical approach to carcinogenicity assessment is to focus on the chemicals of greatest concern, and use human-relevant testing methods to guide the most appropriate risk management measures. The carcinogen potency factor for As, Ni, and Cr-VI all show elevated values, Ni exposures representing the dominant contributor to the total risk of cancer from lifetime consumption of seaweeds, followed by that due to Cr-VI. In respect of the lifetime consumption of seaweed and with it the associated dietary exposure to Cd, Pb, Cr, As, and Ni, a total carcinogenic risk of 2.94 × 10^−1^ has been calculated. According to the US-EPA [35], while values of LTCR of less than 10^−6^ are considered negligible, between 10^−6^ and 10^−4^ the risks are considered to be tolerable. In the present study, with Pb representing the lowest calculated LTCR beyond tolerable levels, projected exposures from Cr-VI, Cd, As, and Ni are all strongly commensurate with tangible risk of cancer resulting from the lifetime consumption of the sampled seaweed. Note that the deduced values shown in Table 5 are related to the dried material (derived from dry weight). Since usually seaweeds are consumed after water hydration, the estimated risk factors may be somewhat lower in value than the derived ones. Notwithstanding this, another important observation is that these higher values are reflective of elevated cancer slope factors (CSF) for the respective metals, with contributions from industrial activities within the marine environment. These findings indicate a crucial need for detailed study of metal levels in seaweed, not limited to study of the present locations, also in regard to the setting-up of regulations on maximal concentration of metals in seaweed products for Asian countries. Additionally, of interest would be investigation of the efficacy of removal of metals in seaweed, availed by bio-adsorbent techniques [86] conducted in seeking to protect the health of consumers. 

## 5. Conclusions

The presence of a range of essential and nonessential elements (K, Ca, Mg, Pb, Cd, Se, Al, Mn, Cu, Zn, Fe, As, Na, Ni, Cr-VI, Ag, and Si) was detected in cultivated seaweed, collected from three commercial farms in Malaysia. In comparison with the particular essential minerals Cu, F, Mg, Zn, and Mn, relatively high-level concentrations of the essential elements Ca, K, and Na were found in all samples. The potential carcinogenic heavy metals, Pb, Cd, As, Ni, Cr-VI, also showed non-negligible concentrations in the analyzed seaweed. Measured data for most of the metals have been found to be in line with literature data, no comparison being made for Si due to a lack of literature values. Considering the health risks arising from daily consumption of seaweed from the Malaysian locations that have been sampled, these factors give rise to potentially toxic metal levels. The daily intake of the majority of the elements was generally below respective guidance on daily dietary allowances, an exception being for potassium, with levels greater than the recommended range. The noncarcinogenic risk, estimated via the hazard quotient, showed a number of values well beyond unity, pointing to potential adverse health hazards to members of the public via regular long-term consumption of the particular seaweeds. The carcinogenic risk from As, Cd, Ni, and Cr-VI, arising from the ingestion of these seaweeds, is in excess of the acceptable risk of 10^−4^. While these issues certainly need to be addressed, seaweed does form a good source of the total dietary intake of protein/nutrients, consumed regularly by East Asian nations. Since seaweed forms a base to the food web, being probably the main source of nutrients for many invertebrates and fish feeding on them, eventually appearing in the human food web, serious attention needs to be paid to the consumption of seaweed. Moreover, with the potentially carcinogenic elements As, Cd, Ni, Pb, and Cr-VI being present at trace levels in seawater, the seaweed is clearly an indicator of anthropogenic activities contaminating the coastal environment of Malaysia. Therefore, monitoring of heavy metals in the macroalgae may provide useful information on the transfer of potentially toxic metals from abiotic compartments (water and sediments) to humans, also helping in understanding the environmental load of potentially toxic metal releases from anthropogenic activities.

## Figures and Tables

**Table 1:** Certified and recovered values for CRM 7405-a.

Analyte	Total Concentration of Metals in CRM 7405-a (mg/kg)		% Recovery
	Certified Value	Recovered	
K	47,500 ± 700	46,550 ± 686	98.0
Ca	15,200 ± 300	15,048 ±297	99.0
Mg	6790 ± 100	6620 ± 97.5	97.5
Pb	0.43 ± 0.03	0.45 ± 0.04	104.7
Cd	0.79 ± 0.02	0.75 ± 0.03	94.9
Al	147 ± 7	141 ± 6	95.9
Mn	14.1 ± 0.7	14.5 ± 0.6	102.8
Cu	1.55 ± 0.07	1.52 ± 0.06	98.1
Zn	13.4 ± 0.5	13.1 ± 0.4	97.7
Fe	311 ± 11	317 ± 8	101.9
As	35.8 ± 0.9	35.26 ± 0.88	98.5
Na	16,200 ± 200	16,038 ± 198	99.0
Ni	2.2 ± 0.1	2.3 ± 0.08	104.6
Cr	3.4 ± 0.1	3.29 ± 0.09	96.8

**Table 2:** The calculated limit of detection (LoD) for the different analytes used in this study.

Analyte	Spectral Line Wavelength (nm)	SD (Blank)	Number of Calibration Standards	Slope of Calibration Curve, m	LoD = 3 × SD(B)/M (µg/kg)
K	766.49	33.65	7	881.4	114.53
Ca	317.933	29.53	7	10,700	8.28
Mg	285.213	38.01	7	25,990	4.39
Pb	220.353	10.76	3	2853	11.31
Cd	228.802	15.22	3	21,210	2.15
Se	196.026	5.25	3	1077	14.62
Al	396.153	14.36	7	13,990	3.08
Mn	257.61	9.394	3	23,490	1.20
Cu	327.393	58.6	3	93,150	1.89
Zn	206.2	3.9	3	13,140	0.89
Fe	238.204	2.51	7	7492	1.01
As	188.979	4.67	3	965.5	14.51
Na	589.592	13.6	7	4427	9.22
Ni	231.604	7.95	3	12,110	1.97
Cr	267.716	10.21	3	6573	4.66
Ag	328.068	9.48	3	82,420	0.35
Si	251.611	81.21	7	6421	37.94

**Table 3:** Metal concentrations in microgram per gram in seaweed collected from three Malaysian locations: Langkawi (LKW), Semporna (SPN), and Kota Belud (KBL). Data are presented in terms of mean ± relative standard deviation (RSD), also their range. Overall mean value for samples collected from the Malaysian locations are compared with literature values from similar studies conducted elsewhere in the world.

Metal	Measured Concentrations of Metals in Microgram Per Gram in the Seaweed Samples under This Study				Literature Data in Microgram Per Gram, Together with the Country of Study
	LKW (*n* = 3)	SPN (*n* = 3)	KBL (*n* = 2)	Overall Mean Value	
	Mean ± RSD (Range)	Mean ± RSD (Range)	Mean ± RSD (Range)		
K	95,450 ± 2.10 (93,400–97,950)	79,575 ± 1.95 (52,950–106,200)	6815 ± 0.94 (6133.5–7496.5)	60,613	7900 ± 3900–71,200 ± 20,200 [3], New Zealand; 31,840–115,790 ± 1280 [47], Spain
Ca	1097 ± 1.59 (267–2731)	1929 ± 0.96 (1481–2378)	2357 ± 1.01 (2121.3–2592.7)	1794	8500 ± 4500–15,300 ± 800 [3], New Zealand 3900 ± 170–10,050 ± 50 [47], Spain
Mg	772 ± 2.19 (765–779)	2280 ± 1.34 (742–3818)	549 ± 0.99 (439.2–658.8)	1200	5650 ± 110–11,810 ± 340 [47], Spain
Pb	8.47 ± 5.49 (6.75–11)	3.75 ± 4.56 (2.75–4.75)	10.85 ± 1.16 (9.76–11.94)	7.69	0.1–12.1 + 4.0 [48], UK; <LOD–6.96 [31], China; 0.14 ± 0.02–1.83 ± 0.99 [3], New Zealand; 8.38–159.39 [49], Egypt 0.003 [50], Turkey 9.5–19.0 [51], Greece 1.31–2.33 [52], Syria 108.54–333.60 [53], Italy
Cd	1.73 ± 1.66 (1.6–1.95)	1.7 ± 3.29 (1.4–2)	1.45 ± 0.46 (1.23–1.67)	1.63	0.23–1.34 [49], Egypt; 0.002–6.4 [31], China; 0.02–10.03 + 4.12 [48], UK 0.011–0.18 [50], Turkey 0.8–3.1 [51], Greece <0.1–<0.5 [52], Syria 5.22–9.58 [53], Italy
Se	4.9 ± 7.36 (4.3–5.5)	33.95 ± 8.89 (6–61.9)	36.85 ± 2.04 (33.18–40.55)	25.2	0.07 ± 0.03–0.17 ± 0.02 [3], New Zealand <LOD‒12 [31], China
Al	69.2 ± 4.13 (47.6–98.6)	18.55 ± 3.28 (16.7–20.4)	36 ± 0.76 (32.4–39.6)	41.3	0.173–4505 [31], China
Mn	13 ± 4.13 (6.5–25.6)	3.35 ± 2.68 (2.5–4.2)	4.7 ± 0.85 (3.53–5.88)	7.02	3.7 ± 0.2–192.3 ± 142.1 [3], New Zealand 0.404–407 [31], China 18.8 + 2.2–778.4 + 38.8 [43], UK <5.0–55.0 ± 1.1 [47], Spain
Cu	2.38 ± 1.86 (1.8–2.9)	1.225 ± 1.16 (0.85–1.6)	1.3 ± 0.83 (0.91–1.70)	1.64	0.47–65.72 [49], Egypt <LOD–39.2 [31], China 4.8+2.0–50.6+10.6 [48], UK 5.09 ± 3.83–23.62 ± 16.03 [3], New Zealand <5.0 [47], Spain
Zn	18.2 ± 2.29 (15.7–20.75)	13.875 ± 1.14 (12–15.75)	22.3 ± 0.62 (13.4–31.2)	18.1	4.95–111.70 [49], Egypt 12.9 + 0.8–1015.5 + 54.4 [48], UK 9.9 ± 0.2–61.0 ± 22.7 [3], New Zealand 17.4 ± 0–71.4 ± 1.3 [47], Spain
Fe	283 ± 1.94 (232.5–324.2)	160 ± 2.77 (133–187)	239 ± 1.74 (215.1–262.9)	227	65.0 + 4.1–1208 + 98.2 [48], UK 13.7 ± 1.8–1227 ± 522 [3], New Zealand 32.9 ± 5.4–103 ± 4.1 [47], Spain 72.38–3865.96 [49], Egypt
As (III)	1.91 ± 6.47 (1.35–2.25)	6.3 ± 2.86 (4.7–7.9)	5 ± 7.75 (4.3–5.7)	4.40	1.88 ± 0.63–51.32 ± 6.49 [3], New Zealand 0.185–71 [31], China 0.10–1.47 [45], China
Na	4286 ± 1.74 (4227–4396)	2375 ± 2.22 (1576–3174)	31.18 ± 1.03 (28.06–34.3)	2231	36,270 ± 1150–17,640 ± 1660 [47], Spain 1700 ± 600–56,700 ± 18,100 [3], New Zealand
Ni	12.97 ± 1.80 (12.3–13.4)	10 ± 1.07 (8–12.75)	10.6 ± 0.25 (6.9–14.3)	10.0	0.3 + 0.1–70.5 + 8.9 ([48]), UK <LOD–8.82 [31], China 3.15–52.56 [49], Egypt
Cr-VI	40.57 ± 1.50 (37–45.8)	30.3 ± 1.75 (23.8–36.8)	40.15 ± 2.27 (32.9–47.37)	26.0	0.8 + 0.1–5.0 + 0.6 [48], UK 0.089–35.7 [31], China 0.44 ± 0.23–2.61 ± 3.08 [3], New Zealand
Ag	0.2 ± 3.96 (0.15–0.25)	0.125 ± 4.88 (0.1–0.15)	0.2 ± 2.84 (0.15–0.25)	0.18	0.9 + 0.2–4.2 + 0.6 [48], UK
Si	391 ± 2.28 (237.7–489.7)	122 ± 5.85 (74–170)	209 ± 1.05 (177.7–240.4)	241	NA

**Table 4:** Projected metal daily intakes in microgram per kilogram per day for a typical East Asian consumer, arising from exclusive consumption of Malaysian seaweed within the diet, together with recommended total dietary intake levels. NA = not available data.

Metal	Daily Intake of Metals (Microgram Per Kilogram/kg/Day)				Recommended Level of Daily Intake of Metals (Microgram Per Gram/kg/Day)
	LKW	SPN	KBL	Mean	
K	14,685	12,242	1048	9325	3510 [74]
Ca	169	297	363	276	800 [75]
Mg	119	351	85	185	350 [76]
Pb	1.30	0.58	1.67	1.2	3.5 [77]
Cd	0.27	0.26	0.22	0.3	1 [77]
Se	0.75	5.22	5.67	3.9	12.5 [77]
Al	10.65	2.85	5.54	6.3	10 [78]
Mn	2.00	0.52	0.72	1.1	5.5 [3]
Cu	0.37	0.19	0.20	0.3	10 [3]
Zn	2.80	2.13	3.43	2.8	45 [77]
Fe	43.5	24.6	36.8	35	8 [79]
As	0.29	0.97	0.77	0.7	10 [80]
Na	659	365	4.8	343	1500 [76]
Ni	2.00	1.54	1.08	1.5	1.0 [80]
Cr-VI	6.24	4.66	1.08	4.0	3 [81]
Ag	0.03	0.02	0.03	0.03	5 [71]
Si	60.2	18.8	32.2	37	NA

**Table 5:** Certified reference doses (R_f_D) and slope factors (SF) of metals, together with noncarcinogenic and carcinogenic risks applied to an East Asian population for whom exclusive consumption of Malaysian seaweed in the diet is assumed. NA indicates data not available. Mirogram/kg/day is equivalent to microgram per gram (μg/g/day).

Metal	Oral Toxicity Reference Dose, R_f_D		Cancer Slope Factor		Estimated Mean Daily Dose (MDD)	Noncarcinogenic Risk	Carcinogenic Risk (LTCR)
	(mg/kg/day)	References	(mg/kg/day)^−1^	References	(mg/kg/day)	Hazard quotient (HQ)	
K	NA	NA			6.39		
Ca	NA	NA			0.189		
Mg	0.140	[36]			0.126	0.903	
Pb	0.004	[33]	0.01	[33]	0.001	0.203	6.08 × 10^−4^
Cd	0.001	[36]	0.38	[87]	2 × 10^−4^	0.171	4.89 × 10^−3^
Se	0.005	[36]			0.003	0.532	
Al	7.00	[32]			0.004	0.001	
Mn	0.046	[88]			0.001	0.016	
Cu	0.040	[36]			2 × 10^−4^	0.004	
Zn	0.3	[41]			0.002	0.006	
Fe	0.700	[88]			0.024	0.034	
As	0.0003	[36]	1.5	[89]	0.001	1.547	5.22 × 10^−2^
Na	NA	NA			0.235		
Ni	0.020	[36]	1.7	[90]	0.001	0.053	1.34 × 10^−1^
Cr-VI	0.003	[36]	0.50	[91]	0.003	0.912	1.03 × 10^−1^
Ag	NA	NA			1.84 × 10^−5^		
Si	NA	NA			0.0254		
*Hazard index (HI) due to all metals in seaweeds, HI = ΣHQc = 4.38*							*ΣLCR = 2.94 × 10^−1^*

## Data Availability

All data are available in the manuscript.
